# Multi-Professional Family Support Programme: A Collective Development at the Neonatal Intensive Care Unit

**DOI:** 10.3390/ijerph21121568

**Published:** 2024-11-26

**Authors:** Bruna Gomes de Souza, Luciana de Cássia Nunes Nascimento, Mirian Fioresi, Lorena Barros Furieri, Flávia Simphronio Balbino, Luísa Maria da Costa Andrade, Maria Edla de Oliveira Bringuente

**Affiliations:** 1 Nursing Division, Cassiano Antônio Moraes University Hospital, Federal University of Espírito Santo, Vitória 29041-295, ES, Brazil; 2 Department of Nursing, Health Sciences Centre, Federal University of Espírito Santo, Vitória 29047-105, ES, Brazil; luciana.nascimento@ufes.br (L.d.C.N.N.); mirian.fioresi@ufes.br (M.F.); lorena.furieri@ufes.br (L.B.F.); maria.bringuente@ufes.br (M.E.d.O.B.); 3 Department of Paediatric Nursing, Paulista Nursing School, Federal University of São Paulo, Sao Paulo 04024-002, SP, Brazil; balbino.flavia@unifesp.br; 4 Center for Health Technology and Services Research, Porto’s Higher School of Nursing, 4200-072 Porto, Portugal; luisaandrade@esenf.pt

**Keywords:** programme development, multi-disciplinary health team, user embracement, family, intensive care units, neonatal

## Abstract

This study was undertaken to structure and validate a Multi-Professional Family Support Programme that was collectively developed at a Neonatal Intensive Care Unit (NICU). This is participative research of the action-research category with a qualitative–quantitative approach conducted at a University Hospital in the southeast of Brazil with the participation of their multi-professional staff. The study was done in four interdependent stages, adapted from the method proposed by Thiollent (2011): organisation, structuring, validation, and diffusion. Qualitative data were analysed following Bardin’s (2016) Content Analysis Technique and presented in categories and sub-categories. The evaluation instruments followed the evaluation criteria proposed by Pasquali (2010). In the analysis of quantitative data, *Cronbach’s Alpha* coefficient was used to verify reliability, *Fleiss’ Kappa* coefficient for measuring agreement, and the *Content Validity Index* for relevance and representativeness. The programme was built collectively and is based on the *Family-Centred Care model*. *Cronbach’s Alpha* reached values above 0.90, which is excellent reliability. There was varying agreement between substantial/perfect and significant (k = 0.68–1.00; *p* < 0.001), and all the evaluation criteria were considered relevant and representative (CVI > 90.0%). The programme and its guiding technologies were structured and validated with high levels of reliability, agreement, relevance, and representativeness.

## 1. Introduction

The Neonatal Intensive Care Unit (NICU) is a hospital area designated for the care of high-risk neonates (NBs) between 0 and 28 days old who require around-the-clock healthcare [1], and it is considered to be a stressful and less than comfortable environment [2]. In the events that result in the hospitalisation of an NB, the family is overwhelmed by feelings of fear, suffering, and insecurity [3] and can feel powerless if they perceive that their role is not being recognised or respected [4].

The Institute for Patient- and Family-Centered Care (IPFCC) outlines that Family-Centred Care (FCC) redefines relationships in healthcare and includes the family in the planning and decision-making of care with professionals and the patient, makes the benefits and risks of the care clear, guarantees quality, and generates comfort in the care [5]; FCC has become the worldwide standard of care in neonatology for more than 20 years, with the family being understood as the primary source of support and strength for NBs [6].

The benefits of the practice of FCC in neonatology are related to a reduction in levels of parental stress [7,8,9,10], the development of parental empowerment [11], an increase in family satisfaction [8,9,12], a gain in NB body weight [8,9], a reduction in the length of hospitalisation [13], increasing adherence to skin-to-skin contact [14], strengthening the family bond with the NB [7,14], and improving the baby’s clinical results [8,12,15] while also improving breastfeeding rates, reducing infection rates, and increasing the parents’ clinical education [8]. A recent study showed a low incidence of severe outcomes (34.4%) in neurological development in very low birth weight NBs in an NICU that adopted the FCC model [15]. Another current study confirmed that FCC can partially mediate the relationship between professional support and parental stress in the NICU, highlighting that emotional, informational, and practical support from health professionals helps parents feel more competent and less overwhelmed, creating a comforting and supportive environment [10]. However, this practice is not yet the condition of most Brazilian institutions, which is confirmed by the difficulties still reported regarding the interaction between the family and the multi-professional team [16]. Other contextual barriers to the practice of FCC were identified in an NICU in Ghana and are related to parental anxiety and stress, inadequate information sharing and education by staff to family members, family culture and religion, inadequate space and logistics to admit other NBs and accommodate families, inadequate staff for the workload, restricted entry of family members into the unit, and negative staff attitudes towards families [17].

Multi-professional programmes for families in the NICU aim to enable parents to cope positively with the stressful situation of their child’s hospitalisation and, based on this condition, to start developing the knowledge, skills, and abilities that will enable them to participate in the basic care of their NB [18].

Highlighted as the main objectives of FCC are the support for the development of a stable and sensitive relationship between parents and children and the acquisition of parenting skills. Both can become more difficult with the hospitalisation of an NB in the NICU, making the creation of programmes for developmental care or Family-Centred Care in neonatology based on individual needs highly relevant [19].

Although FCC is a global standard, its implementation varies. In Brazil, the integration of FCC principles in NICUs faces specific cultural and institutional challenges that this study addresses. The need to change the technical care paradigm in the NICU to a humanised and welcoming culture centred on the family encouraged the realisation of this study, which may benefit not only the family–neonate binomial but all those involved in the process, health professionals, and institutions. Raising the awareness of the multi-professional team in a collective construction, enabling them to provide effective support by changing the organisational culture of the unit, and structuring interventions that are appropriate to the reality of the service based on national and international guidelines will lead to the alleviation and resolution of family needs and demands. This study has the objective of structuring and validating a Multi-Professional Family Support Programme that was collectively developed at a Neonatal Intensive Care Unit. This study’s guiding questions are as follows:

- Will the organisation of a multi-professional group contribute to the construction of a Multi-Professional Family Support Programme in an NICU?
- What strategies should the multi-professional team use when providing family support in an NICU?
- Are the evaluation criteria selected sufficient to validate a Multi-Professional Family Support Programme and its guiding technologies in the NICU?

## 2. Materials and Methods

### 2.1. Study Design

**Figure 1:**
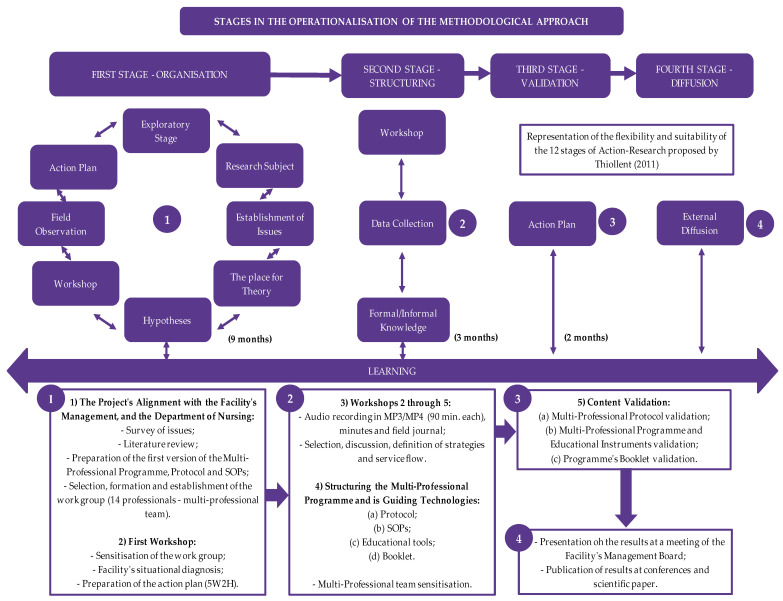
Stages of action-research adapted to interdependent stages.

This is participative research of the action-research category with a qualitative–quantitative approach performed based on the 12 stages proposed by Thiollent [20] and adapted to four interdependent stages: Stage One–Organisation; Stage Two–Structuring; Stage Three–Validation; and Stage Four–Diffusion (Figure 1).

### 2.2. The Research Participants

The participants were 124 professionals, members of the multi-professional staff of the NICU of a University Hospital in the southeast region of Brazil, which belongs to the network of hospitals of the Empresa Brasileira de Serviços Hospitalares (EBSERH). For the “structuring” stage, 14 professionals from the team were intentionally chosen to make up the work group, thus not requiring a sample calculation. The selection criteria included a minimum of five years of experience in neonatal care and active involvement in previous FCC initiatives. The sample size (*n* = 14) was selected to ensure a comprehensive contribution across all relevant professional categories, with data saturation being achieved by the third workshop, with no new themes emerging in subsequent discussions. The work group participated as the experts in the validation stage of the “Multi-Professional Family Support Programme at the Neonatal ICU-Acolher Neo” and its educational technologies (instruments and booklet).

During the “validation” stage, 65 specialists agreed to take part in the validation of the “Multi-Professional Protocol for Family Support at the Neonatal ICU”, all of whom had 2 or more years of experience in neonatology or a degree in the field, as well as having completed a course on support, on the humanisation of neonatal care, or on FCC. The sample size at this stage was calculated using G*Power version 3.1.9.2 [21] to find the optimum effect size [22]: significance level (α = 0.05), power (1-ß = 0.85), constant proportion of acceptance (0.4), resulting in the effect size (d = 0.2), considered to be optimal. Participation was voluntary, and all participants were aware of the purpose of the study, as well as the fact that they could withdraw at any time. The Lead Researcher in this study was the Facilitator throughout the data collection process.

### 2.3. The Research Development

#### 2.3.1. The Project’s Alignment with the Facility’s Management and the Department of Nursing

Stage One, “Organisation”, started in July 2022 and was conducted for 9 months. In order to align the project of the study with sectoral and institutional needs, two meetings were held: the first with the management of the NICU (remote) and the second with the management of the Nursing Department of the facility being analysed (face-to-face). For the survey of problems, three axes were used: 1. Professional Practice; 2. Sectoral Need; and 3. Institutional Need. Subsequently, an integrative literature review was carried out that was developed in 6 stages [23], as well as a survey of documents. The strategies for the support of families in neonatology that were identified were tabulated and categorised according to the principles of FCC (Dignity and Respect; Shared Information; Participation; and Collaboration), and the synthesis of these actions was adapted to the reality of the service according to the activities relevant to each category and the unit’s multi-professional team for later presentation and discussion with the work group. The scientific evidence that emerged from the literature review served as the basis for drawing up the first version of the “Programa Acolher Neo”, a Multi-Professional Protocol and 10 Standard Operating Procedures (SOPs) constructed according to 6 criteria [24]. The establishment of the work group was published in the facility’s service bulletin in March 2023.

#### 2.3.2. First Workshop

The first workshop with the work group was held at a face-to-face meeting at the facility’s premises for a duration of 90 min. The group was sensitised through a group exercise for presentation, self-reflection, and professional critical analysis. The situational diagnosis of the unit was carried out on the basis of a case study and structured according to a roadmap comprising seven stages [25]. The case study was delivered to the participants, who had 10 min to read and identify the problems it contained, which were then discussed by the participants. After the first workshop, the Action Plan was collectively built and validated by the group using the Management Tool 5W2H, and four workshops were established for the collective structuring of the family support systematised model.

#### 2.3.3. Workshops 2 Through 5

Stage Two, “Structuring”, was started in October 2023 and was conducted for 3 months. The workshops were scheduled with a week’s notice and then publicised to the work group using information posters. The second workshop took place face-to-face at the institution, and the last three workshops were held remotely, each lasting 90 min. The number of participants varied from 8 to 12 professionals at each meeting, with all professional categories represented. The workshops were conducted by the facilitator according to a pre-defined agenda, enabling discussions of the scientific evidence identified; the analysis and evaluation of the strategies described and suggested in the first version of the Programme, the Protocol, and the SOPs; determining the best actions and flow of care for family support in a multi-professional manner; and aiming to alleviate and resolve the problems identified during the situational diagnosis. The workshops were audio-recorded in MP3 and MP4 formats, organised into written minutes, and subsequently transcribed and recorded in a field journal [26].

#### 2.3.4. Structuring the Multi-Professional Programme and Its Guiding Technologies

After the last workshop, the scientific evidence identified associated with the data collected with the work group was formatted and described in standardised documents by the institution’s quality department, following the chosen construction and evaluation criteria [24], giving rise to the second version of the “Programa Acolher Neo”, the Multi-Professional Protocol, and the SOPs. The finalised documents were sent to the group via institutional email for consensus evaluation within 15 days. Based on the contributions and suggestions of the work group associated with the service, the need to produce four Educational Instruments was identified (Identification Sign for Team Reference; Affective History; Quiet Time Identification Sign; and Winner’s Certificate), as well as an educational piece of technology of the booklet type, with the programme’s guidelines and routines to be handed out to the families at the NB’s admission at the NICU. The booklet was developed in two adapted stages [27]: 1. Booklet writing; and 2. Booklet editing. Once the “Programa Acolher Neo” and its guiding technologies had been structured, the process of raising the awareness of the entire multi-professional team at the unit began, using six educational posters designed for the production and circulation of information covering relevant topics. Each poster was displayed for a period of 48 h.

#### 2.3.5. Content Validation

The third stage, “Validation”, was conducted for 2 months starting in January 2024. Four forms were drawn up for data collection to validate the “Programa Acolher Neo” and its guiding technologies. The first form enabled the assessment of the multi-professional team’s roles at the four stages of the family’s journey in the NICU, according to the Multi-Professional Protocol: Moment 1—Admission or first family visit to the unit; Moment 2—During the family’s stay at the unit; Moment 3—Discharge from the unit; and Moment 4—Communication of difficult news and support for neonatal grief. The second form was used to evaluate the practical actions of the multi-professional team in the “Programa Acolher Neo”. Ten evaluation criteria adapted from those suggested by Pasquali [28] were used for each question on the forms: Comprehensiveness, Clarity, Coherence, Item Criticality, Objectivity, Scientific Writing, Relevance, Sequence, Unity, and Updated.

The third form, used to evaluate the programme’s four educational tools, was assessed according to four criteria adapted from those proposed by Pasquali [28]: coherence, objectivity, scientific writing and relevance, thus allowing for an assessment of the content in terms of the tools’ language, layout and illustrations. The Programme Booklet was evaluated using the fourth form, structured on the basis of 3 evaluation criteria adapted from those of Pasquali [28]: objectivity (3 questions), scientific writing (4 questions), and relevance (5 questions). A Likert-type scale was used to assess each criterion in all the forms described, with four response scores ranging from 1 (totally disagree) to 4 (totally agree). The qualitative data collected in the seminars directly influenced the refinement of the assessment instruments used for quantitative analysis, ensuring that they were relevant to the needs identified by the working group collaborators. The experts received the products electronically, along with the forms for validation via an electronic form application (*Google Forms*), and had between 10 and 16 days to return the answers, with the invitation being reiterated electronically every three or four days.

### 2.4. Data Analysis

The qualitative data collected in the workshops and described in the field journal were analysed using the Content Analysis Technique according to Bardin [29]. The categories and sub-categories were established according to their relevance and by the resemblance between sentences. Each work group member was identified with the letter “C” in the sequence in which the participation took place (C1, C2, C3, …, C14), and the coding (…) refers to the part of the speech that was omitted.

The quantitative data were analysed using the program IBM SPSS Statistics version 24. The characterisation of these data was presented in the form of observed frequency and percentage. The *Cronbach’s Alpha* coefficient was used to measure the reliability of the forms and between evaluators, with the following cut-off points: less than 0.70 (questionable), 0.70 to 0.80 (acceptable), 0.81 to 0.90 (good reliability), and more than 0.90 (excellent reliability) [30]. The forms were analysed for agreement using the *Fleiss Kappa* coefficient, and the following parameters were used to assess the level of agreement: values below 0.39 (poor), 0.40 to 0.59 (moderate agreement), 0.60 to 0.79 (substantial agreement), and above 0.79 (almost perfect agreement) [31]. The Content Validity Index (CVI), which measures the relevance and representativeness of the instrument through the items in relation to the content of the study, was calculated according to the sum of the answers with values 3 (agree) and 4 (totally agree) divided by the total number of answers [32]. A minimum value of 80.0% was used for validation [33].

### 2.5. Ethical Appraisal

This research was assessed and approved by the Research Ethics Committee under Opinion Number 5.791.291. The experts participated in the research after signing the Informed Consent Form (ICF), which ensures confidentiality and anonymity.

## 3. Results

### 3.1. Work Group Participants and Experts Characterisation in the Process of Content Validation

The work group’s participants and experts who took part in the content validation process were mainly female (over 90%), with more than 30% being nurses with postgraduate qualifications (71.44% and 58.46%, respectively). Detailed demographic characteristics are presented in Table 1.

### 3.2. Comments of the Working Group’s Collaborators on the Analysis of the Field Journal’s Content

Based on the situational diagnosis of the NICU, the working group identified six problems that limited the multi-professional team’s ability to provide effective family care in the unit. The best strategies for Family-Centred Care for hospitalised NBs were identified, analysed, and established in the workshops with the aim of solving these problems. The participants’ reflections on the multi-professional strategies in neonatal family support and the structuring of the “Programa Acolher Neo” were grouped into ten main categories: 1. Parents support and counselling at the NICU (GAAP-Neo); 2. Medical report; 3. Involving the family in decision-making; 4. Inclusion of the family in NB care; 5. Therapeutic workshops; 6. Preparation for hospital release; 7. Family needs assessment; 8. Multi-professional reference team; 9. Communicating difficult news and supporting neonatal grief; 10. Light and noise control. The results of the content analysis of the field journal are presented in Table 2.

The first problem highlighted was the ineffective communication between the multi-professional team and the neonate’s family, and the group discussed two strategies to mitigate this, resulting in the first two central categories. The first category, “Support and Counselling Group for Parents in the NICU (GAAP-Neo)”, was divided into two sub-categories: “How the group was designed and run by the multi-professional team”, and “Affective bond as a facilitator of family adhesion to the group”. The GAAP-Neo group was conceived with the aim of providing the NB’s family with a space to listen, reflect, deal with doubts and feelings, exchange experiences, and offer educational information in a multi-professional way, thus strengthening the bond and trust between the family and the unit’s staff and facilitating the communication process during the hospitalisation period.

Two sub-categories emerged from the second category of “Medical Report”: “Difficulties in routinely completing the medical report” and “Strategy for strengthening the bond between the family and the medical practice”. Given the need to reorganise the medical report in the NICU, a specific time was set (10 a.m.–12 p.m.) for more complete and impartial information to be provided by the unit’s routine and residents doctors to family members, helping to strengthen the bond between those involved and the continuity of information and conduct with the NB.

The second problem identified by the working group was postponed motherhood and fatherhood, which is impacted by the neonate’s hospitalisation, highlighting four multi-professional actions to resolve the problem, with the emergence of categories three through six. The third category, “Involving the family in decision-making”, had the following sub-categories: “The benefits of involving the family in decision-making” and “Affective history”. The Affective History was constructed to facilitate the involvement of the family in decision-making about care planning and the individualised routine of each NB in a shared manner, encouraging the protagonism of the family in a supportive and humanised way. In association with the previous category comes the fourth category, “Involving the family in newborn care”, with the following sub-categories: “Difficulties in involving the family in care” and “Strategies for involving the family in care”. Some of the group’s participants identified weaknesses in this daily practice at the unit, leading to the disconnection of the NB’s care from the family, whether due to work overload, insufficient professional staffing, lack of interest, or maternal absence from the unit. Strategies include reinforcing the importance of family proactivity individually and during workshops and support groups and improving the electronic history of offers and refusals of care.

The fifth category, “Therapeutic workshops”, included the sub-category “The benefits of therapeutic workshops in the development of motherhood”. The group identified that therapeutic activities (manual, expressive, playful, bodily, and educational), when carried out in accordance with the family’s needs, can assist in maternal mental health care, which benefits the collaborative process, their stay in the unit, and the affective bond. The sixth category, “Preparation for hospital discharge”, included the sub-category “Winner’s certificate for the newborn and family”. The Winner’s Certificate was created for the family at the NB’s discharge from the hospital, with the aim of offering the families at the NICU a memento that showed they had overcome the process of hospitalisation and that the multi-professional team was supportive and caring towards the NB and their family.

The third problem that the work group reported was professional insecurity regarding the support to families at the unit, even in the face of the need for daily practice, giving rise to the seventh category, “Assessment of family needs”, and its three sub-categories: “Difficulties in identifying family needs”, “Benefits of targeted and systematised support”, and “Strategies for the applicability of and adherence to the instrument for assessing family needs”. Directing assistance to the family according to their needs is in line with the FCC model; therefore, the instruments “Family Needs in the Neonatal Intensive Care Unit -NEFAM-UTIN” and “Family Needs Addressed in the Neonatal Intensive Care Unit-NEFAT-UTIN” were constructed based on the main needs of the family of an NB hospitalised in the NICU and adapted from those described by Cruz et al. [34] due to the scarcity of instruments capable of mediating the assessment of these needs in the literature. NEFAM-UTIN will help the multi-disciplinary team to identify the relevance of meeting each family’s needs after the NB has been hospitalised, leading to a more systematic and efficient welcome. At the time of hospital discharge, NEFAT-UTIN will enable the verification of the level of family satisfaction with the way their needs were met by the staff. Difficulties such as the psychological condition and understanding of the family during the neonate’s hospitalisation were reported by the group’s collaborators, and strategies were defined for better adherence and applicability of the instrument, such as the family completing it themselves, appointing a facilitator for any family that shows difficulties in this process, and making a QR Code available for digital completion.

The disarticulation of the multi-professional team emerged as the fourth problem highlighted by the group, involving the eighth category, “Multi-professional reference team”, and two sub-categories: “Multi-professional accountability” and “Failure in professional identification”. One of the suggestions for implementation in the unit is the establishment of a “Multi-Professional Reference Team” for NBs and their families, enabling the multi-professional team responsible for each bed to provide continuity in guidance, in the communication process, in the exchange of information, and in the creation of an emotional bond. The group expressed concern about the lack of professional identification in the NICU, and a “Reference Team” nameplate was produced for each box as a facilitating, safe, and supportive tool for families.

The fifth problem identified was inadequate communication of difficult news and grief support, resulting in the ninth category, “Communication of difficult news and support for neonatal grief”, and its sub-categories: “Difficulties in communicating difficult news and privacy” and “Actions to support neonatal grief”. The absence of a room for family assistance in communicating difficult news was highlighted by the participants, who reported a pre-installed normality to the structural deficit, which was generated by the strategies used (empty box and availability of chairs) in an attempt to alleviate the existing problem and ensure a minimum of privacy for the families cared for in the unit. The support actions established for neonatal grief were related to family privacy, providing opportunities for contact with the NB, psychological support, offering an NB’s memory box, and referring interested families to the university’s grief support group called “AcolheDor”.

Inadequate ambience was the sixth and final problem reported by the work group, leading to the tenth category, “Light and noise control”, which is divided into two sub-categories: “Definition of Quiet Time” and “Barriers for carrying out Quiet Time”. The implementation of this intervention will enable better control of light and noise, achieving greater tranquillity in the environment and providing the NB with a resting period (1 h, three times a day) without interruptions. The pre-established timetables for care procedures, fragility in the care grouping, ineffective communication between the staff, a deficit in the professional’s work schedule, and work overload were pointed out by the participants as possible barriers to the realisation and maintenance of the “Hora do Psiu” (Quiet Time). As a result, the role of “Silence Keeper” (a professional who is a reference in controlling noise and light reduction in the NICU) was established, and a sector nameplate was designed for “Quiet Time” with the aim of sensitising and alerting the multi-professional staff and family members present in the unit.

As a result of the strategies established with the work group, the first Multi-Professional Family Support Programme was structured in the neonatal ICU of the institution under study with the definition and standardisation of routines, as well as care flows. The following guiding technologies were also constructed for the programme: Multi-Professional Protocol for Family Support at the Neonatal ICU; SOPs (1. Bad news communication; 2. Efficient communication; 3. GAAP-Neo; 4. Quiet Time; 5. Including the family in decision-making; 6. Involving the parents in the care; 7. Preparation of parents for hospital release; 8. First visit of the parents; 9. The visit of the grandparents; 10. Supervised visit of siblings); forms (NEFAM-UTIN and NEFAT-UTIN); educational tools (1. “Reference Team” sign; 2. Affective History; 3. Quiet Time sign; 4. Winner’s Certificate); and the booklet with guidance from the programme “Acolher Neo”.

### 3.3. “Programa Acolher Neo” and Its Guiding Technologies Content Validation

**Figure 2:**
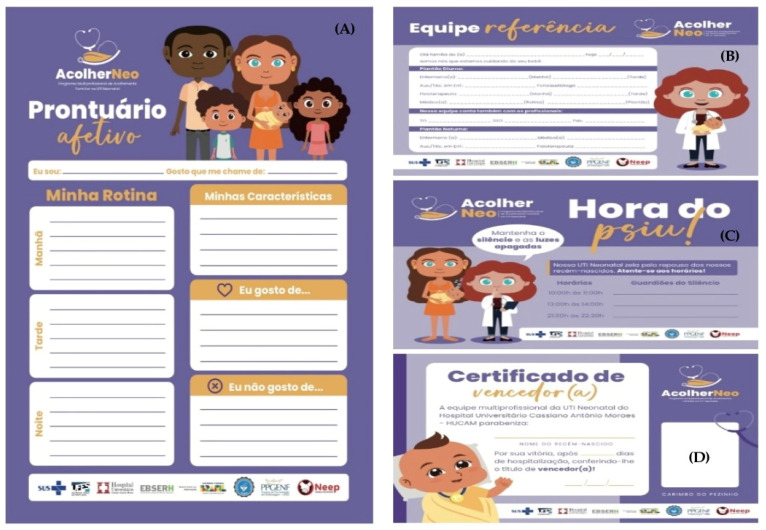
“Acolher Neo” programme’s Educational Instruments. Partial translation from Portuguese and description of text in Figure 3: (**A**) Affective History; name, how I like to be called; my routine; my characteristics; What I like; What I dislike; Morning, Afternoon, Evening; (**B**) Reference Team [space for data of professionals responsible for the care]; (**C**) Quiet Time–Keep silent and the lights off. [timetable with space for the “Quiet Time Guardians” data completion]; (**D**) Winner’s Certificate; The Multi-Professional Team of the Neonatal ICU of the University Hospital Cassiano Antonio Moraes–HUCAM congratulates: ___ NEWBORN’S NAME for their victory, after … days of hospitalisation, granting them the title of winner!

When performing the *Cronbach’s Alpha* Test, it is possible to observe in the evaluation of the reliability of the evaluation criteria of the Multi-Professional Protocol that all achieved values above 0.90 at all moments of the family’s journey in the NICU, as well as in the evaluation of the criteria of the “Acolher Neo” programme, the Educational Instruments, and the Programme Booklet. *Cronbach’s Alpha* values above 0.90 indicate excellent reliability, which means that the instruments used for evaluation were consistently measuring the intended variables. The programme’s Educational Instruments are presented in Figure 2, and the complete results of the evaluation of the reliability of the evaluation criteria are presented in Table 3.

The *Fleiss Kappa* values obtained indicate the existence of agreement among the participants regarding the variables evaluated. The agreement between the evaluation criteria of the Multi-Professional Protocol was almost perfect (above k > 0.80; *p* < 0.001). There was perfect and significant agreement (k = 1.00; *p* < 0.001) between all the criteria assessed in the “Acolher Neo” programme. Among the four Educational Instruments, there was an almost perfect agreement for “Consistency” (k = 0.84; *p* < 0.001) and “Scientific Writing” (k = 0.82; *p* < 0.001). As for the agreement in the evaluation of the Programme Booklet, it was almost perfect and significant (k = 1.00; *p* < 0.001) for “Scientific Writing”. These results are presented in Table 4.

**Figure 3:**
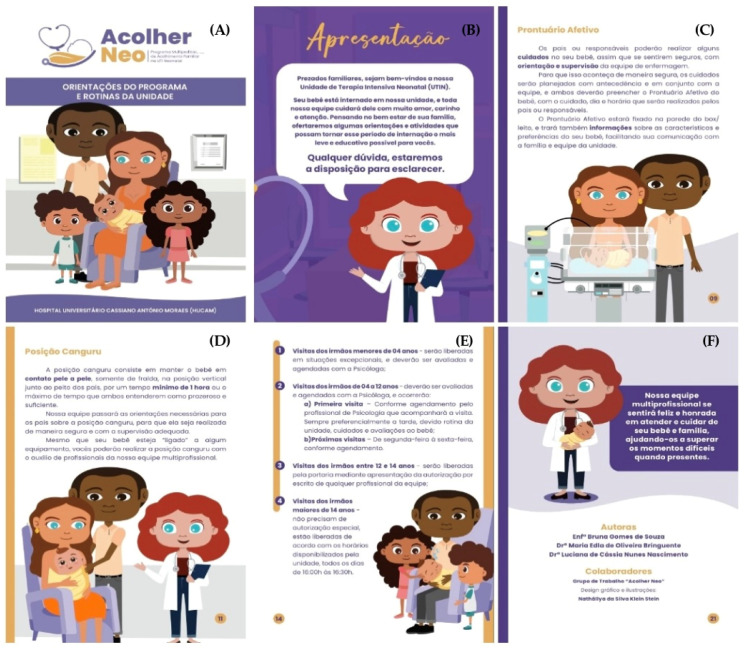
Six of the twenty-two pages of the booklet for the “Acolher Neo” programme. Translation from Portuguese of text in Figure 2: (**A**) Acolher Neo: Multi-Professional Family Support Programme at the Neonatal ICU. GUIDELINES OF THE PROGRAMME, AND UNIT’S ROUTINES. UNIVERSITY HOSPITAL CASSIANO ANTONIO DE MORAES (HUCAM); (**B**) Presentation. Dear Family Members, Welcome to our Neonatal Intensive Care Unit (NICU). Your baby has been admitted to our unit, and our whole team will take care of them with a lot of love, care, and attention. With your family’s well-being in mind, we offer here a few guidelines and activities that may help make this hospitalisation period easier for you and as informative as possible. If you have any questions, we are at your disposal to clarify them. (**C**) Affective History. The parents and guardians will be able to dispense care to your baby as soon as you feel confident about it, with the guidance and supervision of the nursing team. For this to be feasible in a safe manner, the care is planned ahead and with the team, and both should complete the Affective History for the baby, with data about the date and time and what care is being given by the parents or guardians. The Affective History will be affixed on the wall of your box/bed, and it will also have information about your characteristics and preferences regarding your baby, enabling your communication with the family and the unit’s staff. (**D**) Kangaroo Position. The Kangaroo position is one in which the baby is kept with only their diapers on, in contact with the skin, in the vertical position over the breast of a parent for a minimum of 1 h, or the maximum time both understand it is pleasurable and sufficient. Our team will give the parents the necessary instructions for the Kangaroo position for it to be done in a safe manner with adequate supervision. Even if your baby is “linked” to a piece of equipment, you are able to do the Kangaroo position with the help of the staff in our multi-professional team. (**E**) 1 Visits of under 4 yo siblings—These will be allowed in exceptional situations, which will be analysed and scheduled by the psychologist; 2 Visits of siblings from 4 to 12 yo—These shall be evaluated and scheduled by the psychologist, who will supervise the first visit. These preferentially take place in the afternoons due to the unit’s routine and the care and evaluations of the baby. 3 Visits of siblings between 12 and 14 yo—These will be allowed by the reception through the presentation of written authorisation from any of the team’s members; 4 Visits of siblings over 14 yo—These do not need any special authorisation to visit and should occur according to the schedule available for visits at the unit, which is daily from 4 p.m. to 4:30 p.m. (**F**) Our multi-professional team is happy and honoured to assist and care for your baby and your family, supporting you in the difficult moments, if there are any.

As presented in Table 5, all evaluation criteria for the Multi-Professional Protocol reached a CVI higher than 90.0%. In the evaluation of the “Acolher Neo” programme, the criteria reached a CVI of 100.0%. The Educational Instruments achieved a CVI of over 90.0%, with the exception of “Relevance” for Affective History, which obtained 84.62%. All the questions in the Programme Booklet’s evaluated criteria achieved a CVI of over 90.0%. It should be noted that on several questions, the CVI reached 100.0%. Therefore, all the criteria are considered relevant and representative, as they exceeded the minimum required (80.0%). Six of the twenty-two pages of the Programme’s Booklet are represented in Figure 3.

## 4. Discussion

### 4.1. Action-Research in the “Acolher Neo” Programme’s Structuring

Participatory studies are a multifaceted field of research characterised by different methodological orientations. In this context, the theoretical reference point was action-research according to Thiollent [20], with a multi-professional group aiming to structure and validate a Family Reception programme in an NICU and presenting itself as a significant methodological tool in the face of the challenges of multi-professionalism, allowing theory and practice to be associated in the search of strategies to transform the practical reality of the neonatal family support process. Previous studies that have used action-research associated with the focus group technique and combining qualitative and quantitative data have also facilitated the generation of ideas and problem-solving through the participation of group members [35,36,37]. This allows us to understand that the group, when faced with the challenges of everyday problems in their practice, develops the belief that these phenomena can be tackled and worked on based on the collective construction of jointly developed actions, given the sum of multi-professional competencies.

This study highlights the need for tailored FCC training programs in Brazilian NICUs to address unique cultural sensitivities. Collectively structured, the programme enabled the recognition of the limitations and singularities of the multi-professional team, supporting the possibility of broadening their knowledge and improving their ability to provide safer and more qualified family support through the organisational cultural change of the unit, with the structuring of interventions suited to the reality of the service and based on the practice of FCC, which will allow for the alleviation and resolution of family needs and demands in the process of the NB’s hospitalisation. The successful dissemination of innovations in healthcare is best achieved if a guideline is not just repeated but reinvented by the team and adjusted to their local context [38]. In this way, FCC should employ a multi-disciplinary approach [39] in order to contribute to solving the problems under study in an integrated way.

The “Acolher Neo” programme aims to meet the needs of families at different times during their stay in the NICU, from admission to discharge, helping to improve multi-professional knowledge about the importance of including family support as a unit of care in neonatology. Some studies have developed, implemented, and evaluated programmes targeted at neonatal families using the IPFCC’s recommendations. They seek to ensure family empowerment [13]; the implementation of FCC [16]; the education of parents in care, being the main carers of the NB [8]; zero separation between the NB and their parents while receiving care at the NICU [11]; and the offer of multi-professional support to parents to involve them in care [11].

An important limitation of this study is the lack of direct input from families during the development of the programme, which may affect its practical application in FCC contexts. Future studies should include family perspectives to ensure that the programme fully meets their needs.

### 4.2. Reflections on the Multi-Professional Strategies Defined for the Programme “Acolher Neo”

The multi-professional strategies for the support of families in the NICU that were extracted from the work group’s reflections in the content analysis meet the assumptions of the IPFCC and follow its recommendations, with the aim of providing the healthcare team with a flow of care based on evidence and optimising support and assistance for neonatal families.

One of the main strategies for assertive family support is the use of communication, which was deemed ineffective in the unit by the work group participants. Considering that communication permeates the entire family support process, the absence of this skill can cause problems in the transmission of information, resulting in losses for all involved. NICU parents reported in a study in Sweden that the lack of communication with the team hindered their transition to becoming parents and a family [40].

Losses in parenting were pointed out in the workshops and may be associated not only with poor communication but also with resistance on the part of professionals to involve parents in decision-making and in the care of their NB. Shared decision-making with the family regarding the care of the NB, even when the group identifies its benefits, is perhaps one of the most complex interventions for the multi-professional team since it involves adequate communication, strengthened teamwork, the need for time to be given up in the face of work overload, and environmental and operational issues, as well as the psychosocial reality of those involved; these were problems and difficulties also identified by the participants. Experiences in decision-making about palliative care were reported by parents in French NICUs, who discussed the importance of parental autonomy and responsibility in the decision-making process, highlighted the emotional support they received, emphasised the need to receive clear and honest information, and expressed their desire to provide care for their baby [41], needs which are similar to the strategies defined by the work group to alleviate or resolve some of the problems listed.

The disconnection between family and NB care was reported during the discussion with the group about the existing weaknesses in the inclusion of the family in neonatal care, which is further jeopardised by ineffective communication. Studies on parental experiences in neonatal care have identified the need for fathers to be involved in the care of their infants, thus making them feel more capable of parenting [11,39,40,42]. Providing clear explanations to family members can promote collaboration and empowerment for care [35].

Involving parents in neonatal care early on is the first step in preparing for an NB’s discharge from the NICU, a process facilitated by the collaboration of the multi-professional team, adjusting care schedules, strengthening the bond and trust with family members, providing adequate information, and respecting their concerns. The main characteristics of the process of preparing NB parents for discharge from the NICU are related to continuity and an early start, as well as its recognition as a relational, gradual, and dynamic process that seeks the acquisition of knowledge, skills, confidence, and trust by parents for the safe care of their child at home [43].

By identifying professional insecurity about family support in the unit, the importance of assessing family needs to guide the team towards more effective, singular, and systematised care was expressed. Listening carefully to parental opinions is crucial to understanding individual needs and improving the quality of care [11]. Assessing the needs met by family members enables the verification of satisfaction with the conduct by the multi-professional team during the hospitalisation process, allowing the team to see the possibilities for improving their conduct. FCC interventions as instruments for assessing family needs in the NICU are necessary for targeted documentation and analysis of parental learning and individual support needs and can serve as quality assurance tools [44].

Multi-professional strategies were also established for communicating difficult news and supporting neonatal grief, as this is a challenging process for the entire team in the unit, which, in addition to improving all the weaknesses detected in the situational diagnosis, must maintain mental and emotional balance during the interventions. The results of a study on end-of-life care in NICUs in Ibero-America showed the need not only to establish a protocol in the units but also to ensure that multi-disciplinary strategies are developed to implement that protocol [45].

Although FCC is well established in many developed countries, challenges to fully integrate it in underdeveloped countries, such as Brazil, stem from resource constraints and cultural factors. However, the strategies developed here, such as “Therapeutic Workshops” and “Hora do Psiu”, are adaptable and can improve FCC in NICUs across a variety of healthcare settings.

### 4.3. “Acolher Neo” Programme’s Multi-Professional Validation

The “Acolher Neo” programme and its guiding technologies (Multi-Professional Protocol, Educational Instruments, and Programme Booklet) were validated by the experts with excellent reliability (α > 0.90), with agreement varying between substantial/perfect and significant (k = 0.68 to k = 1.00; *p* < 0.001), and evaluation criteria were considered relevant and representative (CVI > 0.90). The technologies are suitable for use in the health education process and for configuring products aimed at meeting family needs, thus favouring and mediating multi-professional support for families in the NICU.

Studies that validated clinical protocols with specialists showed similar results to the current study, valid and relevant content (CVI > 0.80) [46,47,48], and with almost perfect agreement (k = 0.81). The use of protocols can help to standardise care, employing more effective and safer professional actions in accordance with technical–scientific principles [49].

Results from the validation of two health programmes by experts were similar to the current results in terms of relevance (CVI > 0.80) [50,51] and reliability (α > 0.90), while they differed in terms of minimum agreement (k = 0.38 to k = 1.00) [50]. In the validation of the content of educational technologies in health, equivalent results were also observed, with content classified as relevant (CVI > 0.80) [52,53,54] and with excellent agreement (k = 0.94) using the evaluation criteria adapted from Pasquali [53]. Education through different educational methods can improve the perceived effectiveness of FCC [35]; therefore, developing and utilising these methods helps to support and complement the educational process [43].

Strategies to help the multi-professional team in neonatal family support need to be developed, validated, and implemented; in addition, professionals in this area need to be prepared for this supportive care, going through stages of awareness-raising and continuous training. Providing support to the family is not a simple or easy task since human relationships are complex, but health professionals need to feel prepared and acquire the tools to carry out this practice with excellence.

### 4.4. Study’s Contributions

The study arose from a practical need to provide support for the families of neonatal patients based on a national public policy of humanisation and the recommendations of the IPFCC. The structuring and validation was carried out collectively by a multi-professional team. In this context, collective work takes on a process of re-educating all the subjects and actors involved from the perspective of an institutional need, which has as one of its missions the training of healthcare professionals. Hence, the study makes contributions to teaching, research, and extension.

### 4.5. Study’s Limitations

This study presents the limitation of the target (the families) being absent from the process of the structuring and validation of the programme and its technologies, which does not invalidate the results and their applicability in the neonatal context since methodological strictness was followed, scientific evidence was used, and multi-professional team experts participated.

## 5. Conclusions

This study structured and validated a Multi-Professional NICU Family Support Programme and its guiding technologies with high levels of reliability and agreement, and the contents were considered relevant and representative by the experts involved. The programme was structured to implement FCC in the unit under study, providing evidence-based strategies that allow families to be recognised as essential to the health and development of NBs and guiding and optimising the multi-professional team’s care practice in support of families in the NICU. Throughout this process, the participation of managers and professionals involved in the unit’s multi-professional routine was crucial in linking administrative and care needs and favouring and expanding interprofessional collaboration and accountability.

The challenging work of collective construction with the participation of experts in the development of technical and technological educational products coordinated through a research project was based on an integrative and documentary review and on group discussions that enabled the organisation of the best scientific evidence, which plays an important role in the organisation of the “Acolher Neo” programme. The knowledge of recurring problems common to these family members was discussed, reflected on, and deliberated as a form of challenge to be overcome with humanised support in perspective.

Multi-Professional Programmes to implement FCC in neonatology are mainly aimed at fostering the development of and strengthening the affective bond between the parent–neonate binomial and the acquisition of parenting skills, which are hindered by the NB’s hospitalisation.

It is recommended that the programme be implemented in the unit by the multi-professional work group, which took part in the whole collaborative and constructive process and is aware of its responsibility in carrying out and replicating the proposed activities. To implement the “Acolher Neo” programme in other NICUs, we recommend starting with staff training on the principles of FCC, followed by the phased integration of the programme components, such as the use of “Hora do Psiu” and “Winner’s Certificate”. Potential barriers, such as staff resistance or resource constraints, can be mitigated by involving management from the beginning and ensuring regular feedback sessions throughout implementation. 

The actions of the multi-professional team must take into account the family’s psychological conditions, their level of understanding, the family’s resistance to shared care, the overload of professionals, and the deficient work schedules, all of which can impact the process of effective communication, qualified listening, the creation of an emotional bond, health education, and the acceptance of family doubts and feelings. It is feasible and relevant to develop new technologies based on the FCC model that guide NICU professionals towards efficient family care. Professionals need to be continuously sensitised and trained for the development of their relational abilities. Future research is suggested to evaluate the implementation and the effects of the programme on meeting the families’ needs. Future research should explore longitudinal outcomes of FCC integration in NICUs across diverse socioeconomic settings to validate our findings.

This study is believed to contribute to professional practice and research in the neonatal field as an innovative product in the multi-professional context and by associating collective knowledge with national and international guidelines and providing educational tools, with the benefit of supporting family members of NBs hospitalised in the NICU.

## Figures and Tables

**Table 1:** Work group participants and experts characterisation in the process of content validation.

Participant Characterisation		Participants		Experts	
		*n*	%	*n*	%
Sex:	Female	13	92.86	62	95.38
	Male	1	7.14	3	4.62
Age range:	30–39 y.o.	7	50.00	24	36.92
	40–49 y.o.	5	35.71	26	40.00
	50 y.o. or more	2	14.29	15	23.08
Time in education (completed years):	0–4 years	1	7.14	-	-
	5–9 years	1	7.14	3	4.62
	10–14 years	3	21.44	17	26.15
	15–19 years	6	42.86	20	30.77
	20–24 years	1	7.14	12	18.46
	25 years or more	2	14.28	13	20.00
Higher degree:	High School	-	-	8	12.31
	Graduated	1	7.14	7	10.77
	Post-Graduated	10	71.44	38	58.46
	Master’s Degree	2	14.28	11	16.92
	Doctorate	1	7.14	1	1.54
Has a qualification in neonatology?	No	6	42.86	29	44.62
	Yes	8	57.14	36	55.38
Current occupation:	Social Worker	1	7.14	2	3.08
	Nursing Assistant	-	-	2	3.08
	Nurse	5	35.71	20	30.77
	Physiotherapist	1	7.15	10	15.38
	Speech Therapist	1	7.14	2	3.08
	Physician	2	14.29	5	7.69
	Psychologist	1	7.14	3	4.62
	Nursing Technician	2	14.29	19	29.23
	Occupational Therapist	1	7.14	2	3.08
Work sector:	NICU	13	92.86	59	90.77
	NICU and Maternity	1	7.14	5	7.69
	NICU and Paediatrics	-	-	1	1.54
Clinical practice experience in neonatology (in years):	2–4 years	-	-	5	7.69
	5–9 years	7	50.00	27	41.54
	10–14 years	2	14.29	12	18.46
	15–19 years	2	14.29	5	7.69
	20–24 years	1	7.14	6	9.23
	25–29 years	1	7.14	6	9.23
	30 years or more	1	7.14	4	6.15
Is a member of the facility’s Humanisation Committee?	No	12	85.71	-	-
	Yes	2	14.29	-	-
Have you taken any courses on neonatal support care and humanisation, or Family-Centred Care? Which?	Family-Centred Care course	-	-	1	1.54
	Kangaroo Method awareness course	8	57.14	51	78.46
	Kangaroo Method Tutor course	6	42.86	12	18.46
	Has family in the paediatric context	-	-	1	1.54

**Table 2:** Analysis of the working group’s collaborators’ comments.

Problems Identified	Analysis Categories	Analysis Sub-Categories	Work Group Comments
1. Ineffective communication between team and family	Counselling support group for parents at the NICU (GAAP-Neo)	Preparation and conduction of the group by the multi-professional team	Does multi, and it does a professional turn-around (…) to participate in the creation and conduction (C5).
		Affective bond as an enabler of family adherence to the group	When mothers create a bond, they attend the group more (C5).
	Medical bulletin	Barriers for the medical bulletin by routine	Families often have individual issues that change what their needs are (C11).
		Strategy for the strengthening of the bond between the family and the medical staff	The shift gives information, instructs them to come back the next day (…) to have a bond, we need continuity in information (C7).
2. Postponed and hindered maternity and paternity	Inclusion of the family in decision-making	Benefits of the inclusion of the family in the decision-making process	It can aggregate a lot to the quality of care (…) it is easier to have a family that is the protagonist (C6).
		Affective history	Moment for construction with the family (…) One needs to individualise each baby (C6).
		Barriers for the inclusion of the family in the care	Sometimes we offer to the mother to do the care, but they do not want to (C10).
	Inclusion of the family in the newborn care	Strategies for the inclusion of the family in care	These can be worked on either in groups or individually (C6).
	Therapeutic workshops	Benefits of the therapeutic workshops for maternity development	It is a moment of care with the mother’s mental health (…) she comes back calmer, more patient, and more collaborative (C6).
	Preparation for hospital release	Winner’s Certificate for the newborn and the family	It is a memento. I think it reinforces the logic of overcoming in this process (C4).
3. Professional’s insecurity for family support	Evaluation of the family’s needs	Barriers in the identification of the family’s needs	The family arrives very anxious. They do not understand all that you say (C1).
		Benefits of a directed and systemised care	To be included in the making of decisions (…) it is part of what Family Centred Care is (C8).
		Strategies for the applicability and adherence to the instrument to evaluate family needs	I suggest that we make a *Google Form* with a *QR Code* (C5).
4. Multi-professional team disbandment	Reference multi-professional team	Multi-professional accountability	I think that the responsible person can be established by the box (C1).
		Professional identification failure	We could improve the way we deal with the family (…) to improve our presentation to the family (C8).
5. Inadequate difficult news announcement and neonatal grief support	Inadequate difficult news announcement and neonatal grief support	Barriers for the communication of difficult news and privacy	It is sad, but I am so used to the lack of a place to give assistance that I even don’t (…) it is already normal (C7).
		Neonatal grief support actions	Privacy, contact opportunity, family, support connection, psychological support (C9).
6. Inadequate environment	Light and noise control	Establishment of Quiet Time (Hora do Psiu)	It is a truce on light, sound, touching, and interventions (C6).
		Barriers for the establishment of Quiet Time (Hora do Psiu)	Depending on the shift, and how many children are in ventilation, it all ends up making our assistance to be late (C9).

**Table 3:** Evaluation criteria reliability assessment.

*Cronbach’s Alpha* If the Item Is Excluded										
Evaluation Criteria	Multi-Professional Protocol				“Acolher Neo” Programme	Educational Instruments				Programme’s Booklet
	Moment 1	Moment 2	Moment 3	Moment 4		Reference Team Sign	Affective History	Quiet Time Sign	Winner’s Certificate	
Scope	0.986	0.989	0.993	0.994	1.000	-	-	-	-	-
Clarity	0.986	0.988	0.993	0.994	1.000	-	-	-	-	-
Consistency	0.986	0.988	0.994	0.994	1.000	0.967	0.949	0.976	0.957	
Criticality of items	0.986	0.989	0.994	0.993	1.000	-	-	-	-	-
Objectivity	0.986	0.988	0.994	0.993	1.000	0.967	0.949	1.000	0.957	0.952
Scientific Writing	0.987	0.988	0.993	0.993	1.000	0.967	0.978	0.976	1.000	1.000
Relevance	0.986	0.988	0.994	0.993	1.000	1.000	0.971	0.976	0.957	0.962
Sequence	0.986	0.988	0.993	0.993	1.000	-	-	-	-	-
Unity	0.986	0.988	0.994	0.993	1.000	-	-	-	-	-
Updated	0.987	0.989	0.994	0.994	1.000	-	-	-	-	-
Alpha with all items	0.988	0.989	0.994	0.994	1.000	0.981	0.971	0.986	0.977	0.942

Note: The moments refer to the family’s journey at the neonatal ICU.

**Table 4:** Evaluation criteria reliability assessment.

Technologies	Evaluation Criteria	k	Standard Error	*p* Value *	CI of 95% for k	
					Lower Limit	Higher Limit
Multi-Professional Protocol	Scope	0.83	0.04	<0.001	0.75	0.92
	Clarity	0.81	0.04	<0.001	0.73	0.90
	Consistency	0.84	0.04	<0.001	0.76	0.93
	Criticality of items	0.83	0.04	<0.001	0.75	0.92
	Objectivity	0.82	0.04	<0.001	0.73	0.90
	Scientific Writing	0.84	0.04	<0.001	0.76	0.93
	Relevance	0.81	0.04	<0.001	0.73	0.90
	Sequence	0.84	0.04	<0.001	0.76	0.93
	Unity	0.84	0.04	<0.001	0.76	0.93
	Updated	0.85	0.04	<0.001	0.77	0.94
Programme “Acolher Neo”	All 10 criteria	1.00	0.04	<0.001	0.92	1.08
Educational Instruments	Consistency	0.84	0.10	<0.001	0.64	1.04
	Objectivity	0.76	0.10	<0.001	0.57	0.96
	Scientific Writing	0.82	0.11	<0.001	0.62	1.03
	Relevance	0.75	0.09	<0.001	0.57	0.94
Programme Booklet	Objectivity	0.857	0.174	<0.001	0.515	1.198
	Scientific Writing	1.000	0.096	<0.001	0.812	1.188
	Relevance	0.800	0.090	<0.001	0.623	0.975

(*) *Fleiss Kappa* statistics; k-coefficient; significant if *p* ≤ 0.050.

**Table 5:** Evaluation criteria relevance and representativity assessment.

Evaluation Criteria	Multi-Professional Protocol				“Acolher Neo” Programme	Educational Instruments				Programme Booklet
	Moment 1	Moment 2	Moment 3	Moment 4		Reference Team Sign	Affective History	Quiet Time Sign	Winner’s Certificate	
	%	%	%	%	%	%	%	%	%	%
Scope	93.85	93.85	93.85	92.31	100.00	-	-	-	-	-
Clarity	93.85	95.38	95.38	93.85	100.00	-	-	-	-	-
Consistency	92.31	95.38	95.38	92.31	100.00	100.00	92.31	92.31	100.00	-
Criticality of items	95.38	95.38	93.85	93.85	100.00	-	-	-	-	-
Objectivity	93.85	95.38	93.85	93.85	100.00	100.00	92.31	92.31	100.00	100.00
Scientific Writing	93.85	95.38	95.38	93.85	100.00	100.00	100.00	92.31	100.00	90.91
Relevance	93.85	95.38	95.38	93.85	100.00	92.31	84.62	92.31	100.00	100.00
Sequence	92.31	95.38	93.85	93.85	100.00	-	-	-	-	-
Unity	95.38	95.38	95.38	93.85	100.00	-	-	-	-	-
Updated	93.85	95.38	93.85	93.85	100.00	-	-	-	-	-

Note: The moments refer to the family’s journey at the neonatal ICU.

## Data Availability

The data presented in this study are not publicly available due to privacy concerns.

## References

[1] Duarte S.C.M., Azevedo S.S., Muinck G.C., Costa T.F., Cardoso M.M.V.N., Moraes J.R.M.M. (2020). Best Safety Practices in nursing care in Neonatal Intensive Therapy. Rev. Bras. Enferm..

[2] Stübe M., Rosa M.B.C., Pretto C.R., Cruz C.T., Morin P.V., Stumm E.M.F. (2018). Stress in parents of newborns in a Neonatal Intensive Care Unit. Rev. Rene.

[3] Zanfolin L.C., Cerchiari E.A.N., Ganassin F.M.H. (2018). Difficulties Experienced by Mothers during the Hospitalization of their Babies at Neonatal Units. Psicol. Ciência Profissão.

[4] Sanders M.R., Hall S.L. (2018). Trauma-informed care in the newborn intensive care unit: Promoting safety, security and connectedness. J. Perinatol..

[5] (2024). Institute for Patient- and Family-Centered Care. http://www.ipfcc.org/about/index.html.

[6] Moore K.A.C., Coker K., Dubuisson A.B., Swett B., Edwards W.H. (2003). Implementing potentially better practices for improving family-centered care in neonatal intensive care units: Successes and challenges. Pediatrics.

[7] Toivonen M., Lehtonen L., Löyttyniemi E., Ahlqvist-Björkroth S., Axelin A. (2020). Close Collaboration with Parents intervention improves family-centered care in different neonatal unit contexts: A pre–post study. Pediatr. Res..

[8] Zhang R., Huang R., Gao X., Peng X., Zhu L., Rangasamy R., Latour J.M. (2018). Involvements of Parents in the Care of Preterm Infants: A Pilot Study Evaluating a Family-Centered Care Intervention in a Chinese Neonatal ICU. J. Pediatr. Crit. Care Med..

[9] De Bernardo G., Svelto M., Giordano M., Sordino D., Riccitelli M. (2017). Supporting parents in taking care of their infants admitted to a neonatal intensive care unit: A prospective cohort pilot study. Ital. J. Pediatr..

[10] Loutfy A., Zoromba M.A., Mohamed M.A., El-Gazar H.E., Andargeery S.Y., El-Monshed A.H., Belkum C.V., Ali A.S. (2024). Family-centred care as a mediator in the relationship between parental nurse support and parental stress in neonatal intensive care units. BMC Nurs..

[11] Hoogen A.V.D., Eijsermans R., Ockhuijsen H.D.L., Jenken F., Maatman S.M.O., Jongmans M.J., Verhage L., van der Net J., Latour J.M. (2021). Parents’ experiences of VOICE: A novel support programme in the NICU. Nurs. Crit. Care.

[12] Maria A., Litch J.A., Stepanchak M., Sarin E., Wadhwa R., Kumar H. (2021). Assessment of feasibility and acceptability of family-centered care implemented at a neonatal intensive care unit in India. BMC Pediatr..

[13] Beheshtipoor N., Shaker Z., Edraki M., Razavi M., Zare N. (2013). The Effect of family-based empowerment program on the weight and length of hospital stay of preterm infants in the neonatal intensive care unit. GMJ.

[14] Leal L.B., Mathiolli C., Lago M.T.G., Zani A.V. (2021). Paternal experiences of premature babies, music therapy and the kangaroo position: Content analysis. Online Braz. J. Nurs..

[15] Lugli L., Pugliese M., Bertoncelli N., Bedetti L., Agnini C., Guidotti I., Roversi M.F., Della Casa E.M., Cavalleri F., Todeschini A. (2024). Neurodevelopmental Outcome and Neuroimaging of Very Low Birth Weight Infants from an Italian NICU Adopting the Family-Centered Care Model. Children.

[16] Balbino F.S., Balieiro M.M.F.G., Mandetta M.A. (2016). Measurement of Family-centered care perception and parental stress in a neonatal unit. Rev Lat.-Am. Enferm..

[17] Abukari A.S., Schmollgruber S. (2024). Perceived barriers of family-centred care in neonatal intensive care units: A qualitative study. Nurs. Crit. Care.

[18] Moreno D.A.L. (2017). Control del Estrés en los Padres de Neonatos Internados en la Unidad de Cuidados Intensivos Neonatales del Servicio de Neonatología—Hospital Nacional Ramiro Prialé Prialé (EsSalud Huancayo) en el Período 2017–2019. Master’s Dissertation.

[19] Hüning B.M., Reimann M., Sahlmen S., Leibold S., Nabring J.C., Felderhoff-Müser U. (2016). Analysis of a Family-centred Care Programme with Follow-up Home-visits in Neonatology—In Times of the Directive from G-BA. Klin. Pädiatrie.

[20] Thiollent M. (2011). Metodologia da Pesquisa-Ação.

[21] Faul F., Erdfelder E., Lang A.-G., Buchner A. (2007). G*Power 3: A flexible statistical power analysis program for the social. Behav. Res. Methods.

[22] Cohen J. (1988). Statistical Power Analysis for the Behavioral Sciences.

[23] Ganong L.H. (1987). Integrative reviews of nursing research. Res. Nurs. Health.

[24] Pimenta C.A.M., Francisco A.A., Lopes C.T., Nishi F.A., Maia F.O.M., Shimoda G.T., Jensen R. (2015). Guia Para Construção de Protocolos Assistenciais de Enfermagem.

[25] Galdeano L.E., Rossi L.A., Zago M.M.F. (2003). Instructional script for the elaboration of a clinical case study. Rev Lat.-Am. Enferm..

[26] Minayo M.C.S., Deslandes S.F., Gomes R. (2016). Pesquisa Social: Teoria, Método e Criatividade.

[27] Echer I.C. (2005). The development of handbooks of health care guidelines. Rev Lat.-Am. Enferm..

[28] Pasquali L. (2010). Instrumentação Psicológica: Fundamentos e Práticas.

[29] Bardin L. (2016). Análise de Conteúdo.

[30] George D., Mallery P. (2003). SPSS for Windows Step by Step: A Simple Guide and Reference.

[31] Landis J.R., Koch G.G. (1997). The measurement of observer agreement for categorical data. Biometrics.

[32] Wynd C.A., Schmidt B., Schaefer M.A. (2003). Two quantitative approaches for estimating content validity. West. J. Nurs. Res..

[33] Grant J.S., Davis L.L. (1997). Selection and use of content experts for instrument development. Res. Nurs. Health.

[34] Cruz A.C., Balbino F.S., Gaíva M.A.M., Toso B.R.G.O., Mandetta M.A. (2021). Modelo de cuidado centrado no paciente e na família na unidade de terapia intensiva neonatal. Associação Brasileira de Enfermagem, Sociedade Brasileira de Enfermeiros Pediatras.

[35] Nematifard T., Arsalani N., Nourozi T.K., Fallahi-Khoshknab M., Borimnejad L. (2024). Improvement of family-centered care in the pediatric rehabilitation ward: A participatory action research. Front. Pediatr..

[36] Ford M.K., Roberts S.D., Andrade B.F., Desrocher M., Wade S.L., Kohut S.A., Williams T.S. (2023). Building I-INTERACT-North: Participatory Action Research Design of an Online Transdiagnostic Parent–Child Interaction Therapy Program to Optimize Congenital and Neurodevelopmental Risk. J. Clin. Psychol. Med. Settings.

[37] Jeong H.W., Ju D., Choi M.L., Kim S. (2021). Development and Evaluation of a Preceptor Education Program Based on the One-Minute Preceptor Model: Participatory Action Research. Int. J. Environ. Res. Public Health.

[38] Schuler R., Woitschitzky L., Eiben C., Beck J., Jägers A., Windhorst A., Kampschulte B., Petzinger J., Waitz M., Reimer-van Kilsdonk M.O. (2023). Multi-dimensional assessment of infant, parent and staf outcomes during a family centered care enhancement project in a tertiary neonatal intensive care unit: Study protocol of a longitudinal cohort study. BMC Pediatr..

[39] Abukari A.S., Acheampong A.K., Aziato L. (2022). Experiences and contextual practices of family-centered care in Ghanaian nicus: A qualitative study of families and clinicians. BMC Health Serv. Res..

[40] Petersson M.A., Benzein E., Massoudi P., Wåhlin I., Persson C. (2023). Parents’ experiences of the significance of interpersonal interactions for becoming parents and a family during neonatal intensive care. J. Pediatr. Nurs..

[41] Saint D.K., Lamore K., Nandrino J.-L., Rethore S., Prieur C., Mur S., Storme L. (2024). Parents’ experiences of palliative care decision-making in neonatal intensive care units: An interpretative phenomenological analysis. Acta Paediatr..

[42] Linnéra A., Blomqvist Y.T., Jonsson K., Lilliesköld S., Normana M. (2024). Parental Experiences of Neonatal Care: A Nationwide Study on Determinants of Excellence. Neonatology.

[43] Osório S.P., Salazar A.M. (2023). Preparing Parents for Discharge from the Neonatal Unit, the Transition, and Care of Their Preterm Children at Home. Investig. Educ. Enferm..

[44] Ciupitu-Plath C., Tietz F., Herzberg J. (2021). Parent needs assessment instruments in neonatal intensive care units: Implications for parent education interventions. Patient Educ. Couns..

[45] Buenoa M.T.M., Muñoz C., Rodríguez S., Sola A. (2024). End-of-life care in neonatal intensive care units in Iberoamerica: A look from the nursing perspective. An. Pediatría.

[46] Araújo M.M., Pizato N., Rodrigues L.S., Andrade L.S., Moraes V.D., Carvalho K.M.B., Dutra E.S., Botelho P.B., Gonçalves V.S.S. (2023). Development and Validation of Protocol Based on Brazilian Dietary Guidelines for Adults with Diabetes Mellitus Who Attended Primary Health Care. Int. J. Environ. Res. Public Health.

[47] Tramontt C.R., de Jesus J.G.L., Santos T.S.S., Rauber F., Louzada M.L.C., Couto V.D.C., Hochberg J.R.B., Jaime P.C. (2022). Development and Validation of a Protocol for Pregnant Women Based on the Brazilian Dietary Guidelines. Rev. Bras. Ginecol. Obstet..

[48] Yao L., Tan J., Turner C., Wang T. (2021). Development and validation of a Tai chi intervention protocol for managing the fatigue-sleep disturbance-depression symptom cluster in female breast cancer patients. Complement. Ther. Med..

[49] Costa P.C.P., Brito T.J.L., Silva L.S., Guimarães P.R.B., Nogueira L.A., Kalinke L.P. (2023). Content Validation and Applicability of a Nursing Care Protocol for Burn Victims. ESTIMA Braz. J. Enteros. Ther..

[50] Ahmad H.A., Romli M.H., Salim M.S.F., Hamid T.A., Mackenzie L. (2023). A validity study to consult on a protocol of a home hazard management program for falls prevention among community dwelling stroke survivors. PLoS ONE.

[51] Cordeiro A.L.P.C., Braga F.T.M.M., Mata L.R.F., Mendes K.D.S., Fófano R.C., Dalri M.C.B. (2021). Blended learning program for the development of skills in the aspiration of artificial airways. Rev Lat.-Am. Enferm..

[52] Silva M.F.G.D., Nobre L.N., Silva E.D. (2023). Animated Videos Based on Food Processing for Guidance of Brazilian Adults: Validation Study. Interact. J. Med. Res..

[53] Yoneiama I.C.O., Santana A.B., Leite M.T.C., Avelar A.F.M., Belela-Anacleto A.S.C., Maia E.B.S. (2023). Development of an educational technology on clean intermittent bladder catheterization in children to instruct family members. Texto Contexto Enferm..

[54] Llaguno N.S., Pinheiro E.M., Avelar A.F. (2021). Development and validation of the “Sleep Hygiene for Children” booklet. Acta Paul. Enferm..

